# Zinc Sorption Studies on Pectin-Based Biosorbents

**DOI:** 10.3390/ma10070844

**Published:** 2017-07-22

**Authors:** Agata Jakóbik-Kolon, Krzysztof Mitko, Joanna Bok-Badura

**Affiliations:** Faculty of Chemistry, Silesian University of Technology, Krzywoustego 6, Gliwice 44-100, Poland; krzysztof.mitko@polsl.pl (K.M.); joanna.bok-badura@polsl.pl (J.B.-B.)

**Keywords:** biosorption, pectin, zinc sorption, agar-agar, guar gum

## Abstract

The previously-obtained and characterized hybrid pectin-based beads containing agar-agar and guar gum, as well as sole pectin beads (P, for comparison) were examined for zinc ions sorption and desorption properties. The sorption kinetics and equilibrium in the studied system was described by two kinetic models (pseudo-first- and pseudo-second-order) and two isotherms (Langmuir and Freundlich), respectively. The desorption kinetics and equilibrium was also investigated by applying various inorganic acids (nitric, hydrochloric, and sulfuric acid) of various concentrations. In the case of guar gum additive, no significant change in sorption capacity compared to sole pectin beads was observed (q: 37.0 ± 2.6 and 34.7 ± 2.0 mg/g, respectively). Addition of agar-agar significantly decreased the sorption capacity to 22.3 ± 1.0 mg/g, but stripping of zinc(II) ions from this biosorbent was complete even with very diluted acids (0.01 M). Total desorption of zinc from sole pectin and pectin-guar gum beads required acid solution of higher concentration (0.1 M). Sorption rates for all biosorbents are roughly the same and maximum sorption is achieved after 4–5 h. Obtained results and the advantage of our sorbent’s shape formation ability, make the pectin-based biosorbents interesting alternative for zinc(II) ions removal.

## 1. Introduction

Heavy metals, present in the waste waters originating from various branches of industry and agriculture, are a serious environmental hazard. Heavy metals contaminate ground waters, air, soil and may be accumulated in humans, animals and plants. The most hazardous heavy metals are Cd, Cr, Cu, Hg, Ni, Pb, and Zn. Some of these metals, for instance mercury and cadmium, show only adverse effects, i.e., carcinogenesis or damage to nervous, cardiovascular, or digestive systems; others are essential in small amounts as micronutrients. Zinc is an important element for the living organisms, especially for humans, but in excess it may cause problems with brain, liver, kidneys, pancreas, or prostate [1]. Due to the enormous zinc usage in many branches of the industry, it is introduced into the environment. The concentrations of zinc in wastewaters depend on the origin and may be, for example, 500 mg/L (from electroplating industry) [2], 75 mg/L (from the electroplating industry in Greece) [3], 61.88 mg/L (from production of viscose rayon) [4], 12 mg/L (from a plating plant) [5], 10 mg/L (from a closed mine of zinc and lead) [6], and 6.6 mg/L (rinsing water of degreasing and metal plating) [7]. Since the limits of zinc in wastewater introduced into environment range from 1.5–2.61 [8,9,10], zinc ions removal from waste water is an important issue. 

Among the known methods, such as coagulation or precipitation, reverse osmosis, and electrochemical techniques, the sorption, especially biosorption, is one of the most economical and ecological methods, especially when the impurities concentration is not very high. As biosorbents for heavy metal ions, especially for zinc ions, various by-products or materials of natural origin may be used, e.g., dead and living microorganisms, crab shell, mustard biomass, sawdust, corn silk, *Eucalyptus sheathiana* bark, water lettuce dry biomass, *Moringa* or *Sophora japonica* pods, herbaceous plants or pine bark [11,12,13,14,15,16,17,18,19,20,21,22]. In addition, the low sorbent cost and the variety of biosorbents make the biosorption an interesting alternative method for heavy metal ions removal. 

One of the used materials is pectin biosorbent, which has shown high sorption capacities toward various heavy metals, depending on sorbent preparation, type of pectin, additives, temperature, and pH [23,24,25,26,27,28,29]. The significant advantage of pectin biosorbents are their forming ability, which makes the industrial applications easier than in the case of amorphous biomass. 

In addition, the properties of such pectin biosorbents may be modified using various additives [30,31,32,33]. For example, in our previous studies we proposed the use of polysaccharides, such as agar-agar, arabic gum, tragacanth gum, guar gum, karaya gum, and phospholipids, such as lecithin, as the additives for pectin-based biosorbents for zinc ion removal. We proved that the additives influenced the structure of the obtained biosorbents, as well as their properties, e.g., the swelling index [34]. Taking into account the mentioned properties of pectin-based biosorbents and cost of the applied additives, the biosorbents with agar-agar and guar gum additives were selected for further examination. They show good affinity toward zinc ions in a broad pH range and have swelling index similar to swelling index of sole pectin biosorbent. In addition, these additives may decrease the final price of obtained biosorbents.

In this study, the kinetics and equilibrium of zinc ions sorption on the proposed pectin-agar-agar and pectin-guar gum biosorbents were determined. A detailed batch studies of desorption of zinc ions from our pectin-based biosorbents using three mineral acids of various concentrations were also conducted. This examination is very important and in many studies it is overlooked. In addition, the desorption examinations are necessary for further application of biosorbent in the column studies and in order to evaluate the possibility of pectin-based biosorbent reuse.

## 2. Results and Discussion

### 2.1. Effect of Sorbent Dose on Zinc(II) Ions Sorption

**Figure 1:**
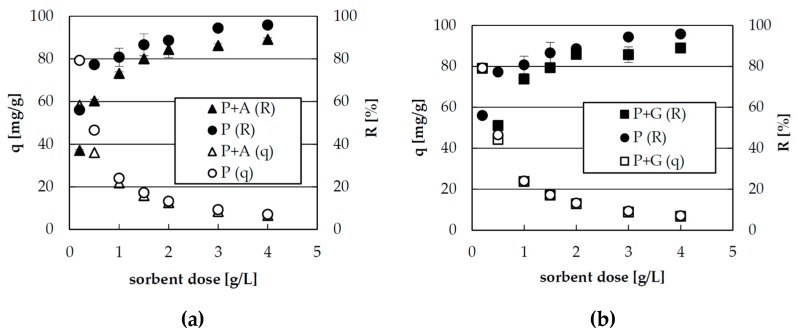
The effect of the sorbent dose on zinc(II) ions sorption on pectin based biosorbents: (**a**) hybrid pectin biosorbent with agar (P+A); and (**b**) hybrid pectin biosorbent with guar gum (P + G). (R) percentage of zinc removal [%], (q) sorption capacity [mg/g].

The effect of sorbent dose on zinc(II) ions sorption is shown in Figure 1. Applying the lowest sorbent dose (0.2 g/L), the highest sorption capacities were obtained (about 80, 80, and 60 for P, P + G, and P + A, respectively), but the percentage of zinc removal in such conditions was significantly lower: about 60, 60, and 40 for P, P + G and P + A, respectively, due to high excess of metal(II) ions. This high excess enabled to fill all the active sites, thus, the sorption capacity at low dose of sorbent was high. At high dose of the sorbent, the majority of metal ions were adsorbed, but some active sites remained unsaturated, which resulted in decrease of the sorption capacity of metal ions per unit mass of adsorbent and increase of percentage of zinc removal. In the experimental conditions, the highest zinc removal was achieved at the sorbent dose ≥ 3 g/L (above 90% for sole pectin and almost 90% for hybrid sorbents), but the sorption capacities were in this case low (7–9 mg/g). At sorbent dose of 1.5 g/L, the zinc removal percentage was still high (above 80%), but the sorption capacity grew twice (almost to 20 mg/g). Therefore, the dose of 1.5 mg/g was chosen and used in further studies. 

### 2.2. Kinetics Studies of Zinc(II) Ions Sorption

**Figure 2:**
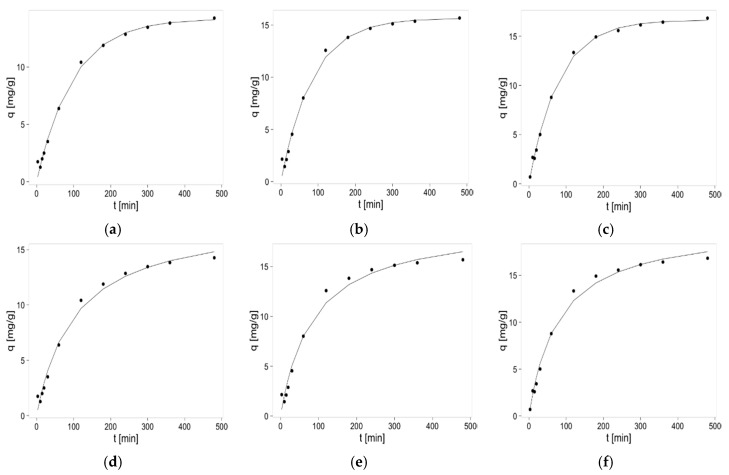
Sorption kinetics of zinc(II) ions on pectin—based biosorbents. Pseudo-first-order model: (**a**) hybrid pectin biosorbent with agar (P + A); (**b**) hybrid pectin biosorbent with guar gum (P + G); (**c**) sole pectin biosorbent (P) and pseudo-second order model; (**d**) hybrid pectin biosorbent with agar (P + A); (**e**) hybrid pectin biosorbent with guar gum (P + G); and (**f**) sole pectin biosorbent (P). Mass of sorbent 0.75 g, initial concentration of metal: c = 30 mg/L, volume of solution: 0.5 L, pH = 6, temperature 22 ± 1 °C.

The parameters of two elementary kinetic models (pseudo-first-order and pseudo-second-order) were estimated based on the results of the Zn(II) sorption kinetics experiments. These parameters and kinetic curves are presented in Table 1 and in Figure 2, respectively. It can be seen that sorption rates for all biosorbents are roughly the same and maximum sorption is achieved after 4–5 h. Due to the complex nature of biosorption, the parameters of kinetic models were calculated mainly to compare various biosorbents in the same, studied conditions, not to settle the sorption mechanism. However, some agreement between the present studies and our preliminary studies concerning these hybrid beads formation and pH-dependent sorption studies may be found. Based on the amount of released calcium ions during preliminary sorption studies, it was stated that in the case of agar-agar addition, probably only Zn-Ca ion-exchange mechanism occurs, in opposite to hybrid biosorbents with guar gum, where physisorption may also take place [34]. The results obtained in current studies for beads with agar additive indicate the pseudo-second-order kinetics (this model fits better to obtained data due to higher R^2^), thus, chemisorption. The results for guar gum additive are quite well described by both models, even better by the pseudo-first-order model, what may indicate mixed sorption mechanism. In summary, the results proved that applied polysaccharide additives do not alter significantly, thus, do not worsen, the sorption kinetics of zinc(II) ions removal on pectin-based beads.

### 2.3. Isotherms of Zinc(II) Ions Sorption

**Figure 3:**
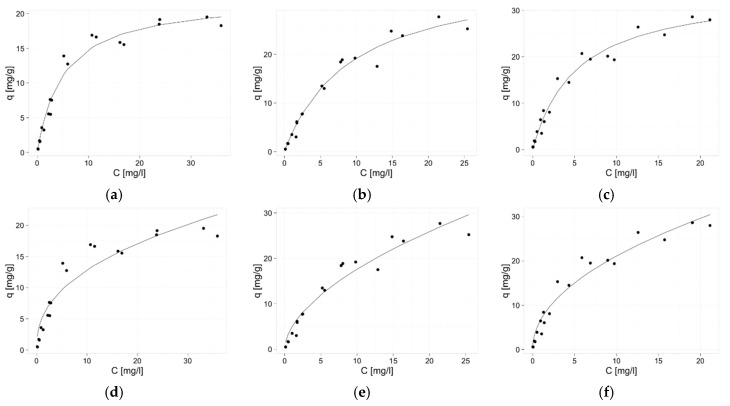
Sorption isotherms of zinc on pectin-based biosorbents. Langmuir model: (**a**) hybrid pectin biosorbent with agar (P + A); (**b**) hybrid pectin biosorbent with guar gum (P + G); (**c**) sole pectin biosorbent (P) and Freundlich model; (**d**) hybrid pectin biosorbent with agar (P + A); (**e**) hybrid pectin biosorbent with guar gum (P + G); (**f**) sole pectin biosorbent (P). Mass of sorbent 0.03 g, volume of solution: 0.02 L, pH = 6, temperature 22 ± 1 °C, time of contact 24 h.

The parameters of two elementary isotherms were calculated based on the results of the Zn(II) equilibrium sorption experiments. These parameters and equilibrium curves are shown in Table 2 and Figure 3, respectively. Since the calculated correlation coefficients are higher for Langmuir than for Freundlich model fitting, the parameters of this model were used for comparison of the sorption properties of studied biosorbents. Addition of agar-agar to pectin beads significantly decreased the sorption capacity, while in the case of guar gum additive, no significant change was observed. Reversed dependence was noticed in the case of B parameter comparison—the adsorption energy, thus, sorption affinity of biosorbent to zinc ions, is almost the same for hybrid with agar and sole pectin biosorbent. The zinc ions affinity of the hybrid sorbent with guar gum is lower by half. The B parameter allows also for calculation of dimensionless constant separation factor R_L_ [35], which indicates character of sorption (R_L_ > 1, unfavorable; R_L_ = 1, linear; 0 < R_L_ < 1, favorable; and R_L_ = 0, irreversible). Obtained results (0.07–0.85, 0.13–0.91, and 0.08–0.85 for P+A, P+G, and P beads, respectively) indicate the favorable sorption in the whole investigated range of zinc(II) initial concentration. However, irreversible adsorption (R_L_ < 0.1) may be proven only for sole pectin biosorbent and biosorbent with agar addition when initial concentration is higher or equal 50 mg/L.

Sorption capacities of our biosorbents are generally higher or similar to the ones reported in the literature for other sorbents of such origin (Table 3), but some biosorbents of higher sorption capacities are also featured (Table 3, labeled with asterisk (*)). However, taking into account the advantage of our sorbent’s shape formation ability, pectin-based biosorbents are interesting alternative for zinc(II) ions removal.

### 2.4. Desorption of Zinc(II) from Biosorbents—Equlibrium

**Figure 4:**
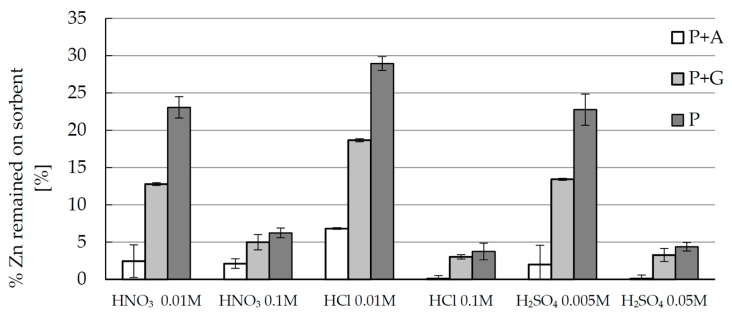
Comparison of zinc(II) ions desorption from pectin-based biosorbents (P + A—hybrid pectin biosorbent with agar, P + G—hybrid pectin biosorbent with guar gum, P—sole pectin biosorbent) using various acid solutions of various concentration. Mass of sorbent 0.03 g, volume of solution: 0.01 L, pH = 6, temperature 22 ± 1 °C, time of contact 24 h.

Desorption is very important issue in the sorption studies because it often prejudges the economic aspects of the process. In order to check the possibility of zinc ions removal from the studied biosorbents and biosorbents reuse, desorption batch studies utilizing various acid solution of various concentration were conducted. The results are shown in Figure 4. The degree of desorption expressed as percent of zinc(II) ions remained on the sorbent after stripping with acid solution differs significantly with biosorbent composition, as well as with acid concentration, and only slightly with kind of acid (the hydrochloric acids seems to be the less efficient desorption medium). The best results were obtained in the case of beads with agar, where desorption was almost complete (>95%) independently of acid kind and concentration. Sorbent with guar gum, as well as sole pectin biosorbent, require usage of more concentrated acid to achieve such a good removal results. However, stripping of zinc ions from the sole pectin biosorbent is the hardest. In the case of applying more diluted acids, independently of the kind of acid, after desorption over 20% of sorbed zinc ions still remains on the sorbent. In summary, it may be stated that applied polysaccharide additives improved significantly the efficiency of desorption. In the case of hybrid pectin-agar biosorbent, ten times less concentrated acid is required to achieve stripping of zinc(II) ions at the level >95% than in the case of sole pectin biosorbent. It may significantly decrease the overall process cost.

### 2.5. Desorption of Zinc(II) from Biosorbents—Kinetics

**Figure 5:**
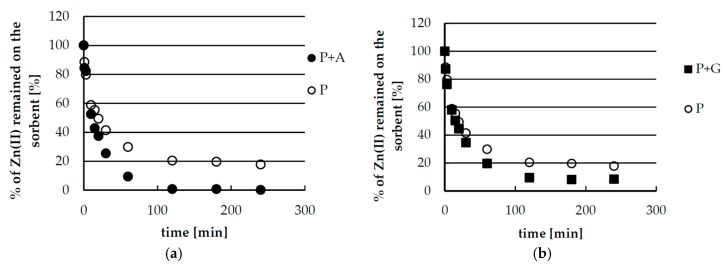

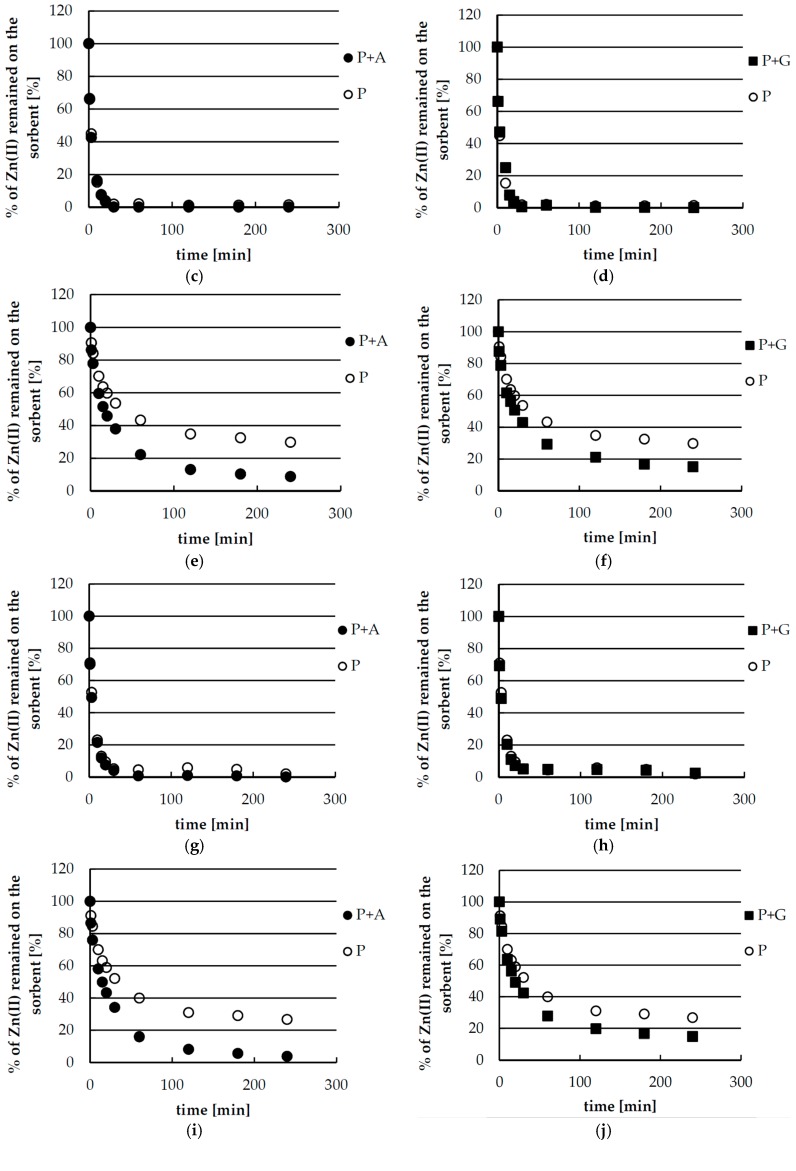

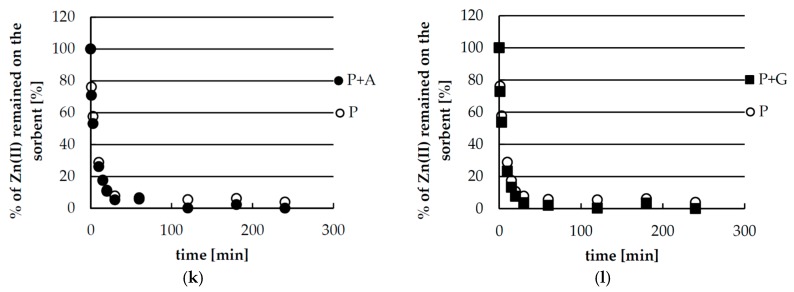
Kinetics of zinc(II) ions desorption from pectin-based biosorbents (P + A—hybrid pectin biosorbent with agar, P + G—hybrid pectin biosorbent with guar gum, P—sole pectin biosorbent) using various acid solutions of various concentration: (**a**,**b**) 0.01 M HNO_3_; (**c**,**d**) 0.1 M HNO_3_; (**e**,**f**) 0.01 M HCl; (**g**,**h**) 0.1 HCl; (**i**,**j**) 0.005 M H_2_SO_4_; and (**k**,**l**) 0.05 M H_2_SO_4_. Mass of sorbent 0.75 g, volume of solution: 0.25 L, temperature 22 ± 1 °C.

Kinetics of desorption is an important factor, especially in the design of the column processes, which are desirable for industrial application. Batch studies showed only slight differences in equilibrium of desorption of zinc depending on kind of acid, especially between nitric and sulfuric acid. Therefore, the kinetic studies were performed to choose the best media for zinc(II) ions desorption from the studied biosorbents. The results are shown in Figure 5. Generally, by using more concentrated acid solutions (0.1 M HNO_3_, 0.1 M HCl, 0.05 M H_2_SO_4_), fast and complete striping of zinc is obtained independently from acid and biosorbent kind. Total zinc removal is achieved in only 30 min. The results also proved the equilibrium limitation observed in the batch studies. More diluted acids (0.01 M HNO_3_, 0.01 M HCl, 0.005 M H_2_SO_4_) allowed for complete removal of zinc(II) ions only from pectin-agar beads. However, usage of such diluted acids required at least 3 h of contact to reach the equilibrium. Desorption was slightly faster for nitric acid and slower for hydrochloric acid. Since these differences are not very significant and differences in the case of more concentrated acids are not significant at all, the selection of the best media for stripping the zinc(II) ions may be limited by other factors, e.g., further methods of metal recovery from the solution.

## 3. Materials and Methods

### 3.1. Materials

The previously obtained and characterized hybrid pectin-based beads containing agar-agar (P + A, P:A = 1:1) and guar gum (P + G, P:G = 1:0.5) [34], as well as sole pectin beads (P, for comparison) were used. The other reagents and standards used for solutions preparation such as: sodium hydroxide (Avantor, Gliwice, Poland), concentrated nitric, sulfuric and hydrochloric acid (all “Suprapur” from Merck, Darmstadt, Germany), zinc nitrate (Avantor, Poland) and standard solutions of zinc and calcium (1000 mg/L, Merck, Germany) were purchased and used without further purification. Millipore Elix 10 system (Milipore SAS, Molsheim, France) was utilized for deionized water preparation.

### 3.2. Analytical Method

The determination of zinc(II) was performed using ICP-AES method (ICP atomic emission spectrometer Varian 710-ES, Varian, Palo Alto, CA, USA). The following parameters were utilized: RF power 1.0 kW, plasma flow 15 L/min, auxiliary flow 1.5 L/min, nebulizer pressure 200 kPa, pump rate 15 rpm, emission lines: λ_Zn_ = 328.233 nm, λ_Zn_ = 334.502 nm, λ_Zn_ = 472.215 nm. Calibration curve method with standard solutions of Zn in the range of 0.1–40 mg/L was used. The thermostated shaker (Incu-Shaker, Benchmark, Sayreville, NJ, USA) was used in sorption-desorption batch studies.

### 3.3. General Procedure for Zinc(II) Ion Sorption Studies

Sorbent samples in dry form were weighed and placed in plastic closed vessels with solution of Zn(NO_3_)_2_ of pH = 6. Next the samples were shaken at room temperature (22 ± 1 °C) for 24 h. After that sample of solution was taken and diluted properly with deionized water in volumetric flask. Concentration of zinc ions in each solution was determined using ICP-AES method. 

The main sorption parameter, sorption capacity of zinc(II) ions on studied pectin-based biosorbents (mg/g), was calculated using the formula:

(1) $$q=\frac{\left(c_{0}-c\right)\times V}{m}\;$$

where:

$c_{0}$—the initial concentration of zinc(II) ions in the solution (mg/L),
c—the final concentration of zinc(II) ions in the solution (mg/L),
V—the volume of the solution (L),
m—the mass of the sorbent (g).

### 3.4. Effect of Sorbent Dose on Zinc(II) Ions Sorption

The studies were performed as described in general procedure (Section 3.3), but 50 mL of solution of initial Zn(II) concentration 30 mg/L and various sample weights (0.01–0.2 g) were used. 

### 3.5. Kinetics Studies of Zinc(II) Ions Sorption

The studies were performed as described in general procedure (Section 3.3), but 500 mL of solution of initial Zn(II) concentration 30 mg/L and 0.75 g of pectin-based beads was utilized. Samples of 0.1 mL were collected after 1, 3, 10, 15, 20, 30, 60, 120, 180, 240, 300, 360, and 480 min. The change of solution volume during experiments caused by sample taking was neglected. This was only 0.26% solution lost during whole experiments. 

The parameters of two elementary kinetic models (pseudo-first-order and pseudo-second-order) were calculated from the results of the Zn(II) sorption kinetics experiments. The following equations were used:

The pseudo-first-order equation [41,42]:

(2) $$\ln\left(q_{m}-q_{t}\right)={\mathrm{lnq}}_{m}-k_{1}\;\cdot \;t$$

The pseudo-second-order equation [42]:

(3) $$t/q_{t}=1/\left(k_{2}\;\cdot \;q_{m}{}^{2}\right)+t/q_{m}$$

where:

q_m_—the zinc(II) ions sorbed on one gram of biosorbent at equilibrium (adsorption capacity) (mg/g),
q_t_—the zinc(II) ions sorbed on one gram of biosorbent at time “t” (mg/g),
k_1_—the rate constant of pseudo-first order sorption model (1/min),
k_2_—the rate constant of pseudo-second order sorption model (g/mg·min),
t—the time (min).

The model parameters were estimated by the least squares method to best fit the data.

### 3.6. Isotherms of Zinc(II) Ions Sorption

The studies were performed as described in general procedure (Section 3.3), but 20 mL of solutions of various concentration (1–60 mg/L) and 0.03 g of biosorbents were used.

The parameters of two elementary isotherms were estimated based on the results of the Zn(II) equilibrium sorption experiments. The following equations were used:

Langmuir isotherm [43]:

(4) $$q=\left(q_{m}\;\cdot \;B\;\cdot \;c\right)/\left(1+B\;\cdot \;c\right)$$

Freundlich isotherm [43]:

(5) $$q=K\;\cdot \;c^{1/n}$$

where:

q—the zinc(II) ions sorbed on one gram of biosorbent at equilibrium (sorption capacity) (mg/g),
q_m_—the maximum sorption capacity (mg/g),
B—the equilibrium constant that corresponds to the sorption energy (L/mg),
c—the equilibrium concentration of zinc(II) ions in the solution (mg/L),
K—((mg/g)(L/mg)^1/n^) corresponds to the relative sorption capacity,
n—corresponds to the sorption intensity of the sorbent.

The model parameters were estimated by the least squares method to best fit the data.

### 3.7. Desorption of Zinc(II) from Biosorbents—Equilibrium

First, the biosorbents were filled with Zn(II) according to general procedure (Section 3.3), but 20 mL of solution of initial Zn(II) concentration 30 mg/L and 0.03 g of pectin-based beads was used. Next, the sorbents were separated from the solution, gently dried with paper towel, and placed in the plastic closed vessels with 10 mL of proper acid solution (0.01 M HNO_3_, 0.1 M HNO_3_, 0.01 M HCl, 0.1 M HCl, 0.005 M H_2_SO_4_, 0.05 M H_2_SO_4_). Next, the samples were shaken at room temperature (22 ± 1 °C) for 24 h. After that, the sample of solution was taken and diluted properly with deionized water in volumetric flask. Concentration of zinc ions in each solution was determined using ICP-AES method. The degree of desorption was calculated using the amount of zinc(II) ions sorbed on the beads during sorption step and amount of zinc(II) ions found in the acid solution after desorption. As the result, the amount of zinc(II) ions remained on the biosorbent (in mass percent) was obtained. 

### 3.8. Desorption of Zinc(II) from Biosorbents—Kinetics

First, the biosorbents were filled with Zn(II) according to general procedure (Section 3.3), but 500 mL of solution of initial Zn(II) concentration 30 mg/L and 0.75 g of pectin-based beads was used. Next, the sorbents were separated from the solution, gently dried with paper towel and placed in the plastic closed vessels with 250 mL of proper acid solution (0.01 M HNO_3_, 0.1 M HNO_3_, 0.01 M HCl, 0.1 M HCl, 0.005 M H_2_SO_4_, 0.05 M H_2_SO_4_). Next, the samples were shaken at room temperature (22 ± 1 °C) and the samples were taken after 1, 3, 10, 15, 20, 30, 60, 120, 180, and 240 min and diluted properly with deionized water in volumetric flasks. Concentration of zinc(II) ions in each solution was determined using ICP-AES method. The degree of desorption was calculated using the amount of zinc(II) ions sorbed on the beads during sorption step and amount of zinc(II) ions found in the acid solution after proper time of desorption. As the result, the amount of zinc(II) ions remained on the biosorbent (in mass percent) at given time was obtained. 

Each experiment was repeated trice, so each result is an average value from three independent trial. 

## 4. Conclusions

Our studies showed that the addition polysaccharides other than pectin to the biosorbents changes some of their sorption properties, especially stripping. In the case of guar gum additive, no significant change in sorption capacity compared to sole pectin beads was observed (q: 37.0 ± 2.6 and 34.7 ± 2.0 mg/g, respectively). Addition of agar-agar significantly decreased the sorption capacity to 22.3 ± 1.0 mg/g, but stripping of zinc(II) ions from this biosorbent was complete even with very diluted acids (0.01 M). Sorption rates of all biosorbents were roughly the same and maximum sorption was achieved after 4–5 h. Since sorption capacities of our pectin-based beads are generally higher, or similar, to the ones reported in the literature for other biosorbents and our biosorbent may be easily formed, such pectin-based biosorbents are an interesting alternative for zinc(II) ions removal.

## Figures and Tables

**Table 1:** Estimated parameters of pseudo-first and pseudo-second order adsorption kinetics model of zinc(II) ions sorption on pectin-based biosorbents (P + A—hybrid pectin biosorbent with agar, P + G—hybrid pectin biosorbent with guar gum, P—sole pectin biosorbent).

Pseudo-First-Order Kinetics			
sorbent	P + A	P + G	P
R^2^	0.944	0.991	0.997
q_m_ [mg/g]	14.2 ± 0.4	15.7 ± 0.4	16.7 ± 0.02
k_1_·10^2^ [1/min]	1.02 ± 0.07	1.19 ± 0.01	1.26 ± 0.05
**Pseudo-Second-Order Kinetics**			
sorbent	P + A	P + G	P
R^2^	0.989	0.982	0.992
q_m_ [mg/g]	18.0 ± 0.8	19.4 ± 1.0	20.4 ± 0.7
k_2_·10^4^ [g/mg·min]	5.41 ± 0.89	6.07 ± 1.19	6.21 ± 0.82

**Table 2:** Calculated parameters of isotherms, Langmuir and Freundlich, of Zn(II) ions sorption on pectin-based biosorbents (P + A—hybrid pectin biosorbent with agar, P + G—hybrid pectin biosorbent with guar gum, P—sole pectin biosorbent).

Calculated Parameters of Langmuir Isotherm			
sorbent	P + A	P + G	P
R^2^	0.973	0.976	0.973
q_m_ [mg/g]	22.3 ± 1.0	37.0 ± 2.6	34.7 ± 2.0
B [L/mg]	0.196 ± 0.027	0.108 ± 0.017	0.190 ± 0.029
**Calculated Parameters of Freundlich Isotherm**			
sorbent	P + A	P + G	P
R^2^	0.913	0.952	0.957
K [(mg/g)(L/mg)^1/n^]	4.97 ± 0.63	4.90 ± 0.57	6.85 ± 0.63
n	2.43 ± 0.26	1.80 ± 0.15	2.05 ± 0.16

**Table 3:** The reported zinc ion sorption capacities of various biosorbents.

Sorbent	Sorption Capacity (mg/g)	Reference
Corn silk	13.98	[15]
Roots of the halophyte	~0.5	[36]
Live biomass fungus Mucor rouxii	7.75	[37]
Carrot residues	29.61	[38]
*Sophora jap*. pods	25.71	[19]
*Typha latifolia* root	28.8	[20]
Seaweed biomass	49.54	[39]
Malaysian Rhodophyte	43.48	[40]
Crab shell *	117	[12]
*Androgaphis paniculata* leaves powder *	86.96	[21]
Modified EUCALYPTUS SHEATHIANA bark *	250	[16]

* biosorbents of substantially higher sorption capacity than ours.
